# Fish oil supplementation suppresses resistance exercise and feeding‐induced increases in anabolic signaling without affecting myofibrillar protein synthesis in young men

**DOI:** 10.14814/phy2.12715

**Published:** 2016-03-23

**Authors:** Chris McGlory, Sophie L. Wardle, Lindsay S. Macnaughton, Oliver C. Witard, Fraser Scott, James Dick, J. Gordon Bell, Stuart M. Phillips, Stuart D. R. Galloway, D. Lee Hamilton, Kevin D. Tipton

**Affiliations:** ^1^ Exercise and Metabolism Research GroupDepartment of KinesiologyMcMaster UniversityHamiltonOntarioCanada; ^2^ Health and Exercise Sciences Research GroupSchool of SportUniversity of StirlingStirlingUK; ^3^ Nutrition GroupInstitute of AquacultureSchool of Natural SciencesUniversity of StirlingStirlingUK

**Keywords:** Fish oil, myofibrillar muscle protein synthesis, p70S6K1, resistance exercise

## Abstract

Fish oil (FO) supplementation potentiates muscle protein synthesis (MPS) in response to a hyperaminoacidemic–hyperinsulinemic infusion. Whether FO supplementation potentiates MPS in response to protein ingestion or when protein ingestion is combined with resistance exercise (RE) remains unknown. In a randomized, parallel group design, 20 healthy males were randomized to receive 5 g/day of either FO or coconut oil control (CO) for 8 weeks. After supplementation, participants performed a bout of unilateral RE followed by ingestion of 30 g of whey protein. Skeletal muscle biopsies were obtained before and after supplementation for assessment of muscle lipid composition and relevant protein kinase activities. Infusion of l‐[*ring*‐^13^C_6_] phenylalanine was used to measure basal myofibrillar MPS at rest (REST), in a nonexercised leg following protein ingestion (FED) and following RE and protein ingestion (FEDEX). MPS was significantly elevated above REST during FEDEX in both the FO and CO groups, but there was no effect of supplementation. There was a significant increase in MPS in both groups above REST during FED but no effect of supplementation. Supplementation significantly decreased panPKB activity at REST in the FO group but not the CO group. There was a significant increase from REST at post‐RE for PKB and AMPK *α*2 activity in the CO group but not in the FO group. In FEDEX, there was a significant increase in p70S6K1 activity from REST at 3 h in the CO group only. These data highlight that 8 weeks of FO supplementation alters kinase signaling activity in response to RE plus protein ingestion without influencing MPS.

## Introduction

Muscle protein synthesis (MPS) is an important metabolic determinant of human skeletal muscle mass (Glynn et al. 2010; McGlory and Phillips 2014). Resistance exercise and provision of a source of essential amino acids (EAAs) are potent stimulators of MPS (Witard et al. 2014). Thus, repeated bouts of resistance exercise and protein feeding result in skeletal muscle hypertrophy (Cermak et al. 2012). The anabolic influence of protein ingestion and resistance exercise on skeletal muscle has led to studies examining the influence of the type (Tang et al. 2009) and dose (Witard et al. 2014) of protein on rates of MPS. Collectively, these studies have shown that in young male adults the consumption of ~0.25 g/kg of high‐quality protein results in the saturation of both rested postprandial (Witard et al. 2014) and postexercise rates of MPS (Moore et al. 2009; Witard et al. 2014) with higher doses of protein resulting in excess urea production and amino acid oxidation (Moore et al. 2009; Witard et al. 2014). The failure of protein doses above 0.25 g/kg to further enhance rates of MPS is related to the inability of the translational machinery to utilize the excess available amino acids for the purposes of protein synthesis, a phenomenon termed the muscle full effect (Bohe et al. 2001; Atherton et al. 2010).

Despite using different doses of amino acids (Moore et al. 2009; Witard et al. 2014) as well as the coingestion of carbohydrate (Staples et al. 2011), no nutritional mechanism has been shown to alter this muscle full effect. However, while the capability of carbohydrate (Staples et al. 2011) and individual amino acids (Churchward‐Venne et al. 2014) to enhance the MPS response to protein ingestion has been studied in detail (Tipton and Phillips 2013), the role of fatty acids in increasing the utilization of ingested protein for the stimulation of MPS has only recently received attention. In this regard, recent studies have demonstrated that supplementation with n‐3 polyunsaturated fatty acid (PUFA)‐enriched fish oil (FO) also confers skeletal muscle anabolic responses. For instance, 8 weeks of FO derived n‐3 PUFA supplementation was shown to potentiate rates of mixed MPS in response to a hyperaminoacidemic–hyperinsulinemic infusion in young, middle‐aged (Smith et al. 2011b), and older adults (Smith et al. 2011a). Additionally, supplementing elderly women with FO during 12 weeks of resistance exercise training has been demonstrated to improve skeletal muscle strength (Rodacki et al. 2012), while one study has shown 6 months of FO supplementation, in the absence of resistance exercise, improves muscle mass and function in elderly men (Smith et al. 2015). Thus, it appears that FO supplementation enhances the n‐3 PUFA composition of skeletal muscle (Smith et al. 2011a, b; McGlory et al. 2014a), which subsequently primes skeletal muscle to respond to anabolic stimulation either in the form of amino acid provision (Smith et al. 2011a, b) or mechanical stimulation (i.e., resistance exercise).

Although there is growing evidence for the efficacy of FO supplementation to enhance muscle anabolism, there remain several practical considerations that need to be addressed. First, the administration of amino acids through an intravenous infusion (Smith et al. 2011a, b) is not a viable means for the general population to consume protein. Second, it is clear that the metabolic response to infusion of amino acids differs from ingestion of an intact protein (Bohe et al. 2001). In the context of an infusion, a square‐wave response of aminoacidemia results in a refractory response such that MPS declines even as aminoacidemia remains constant and elevated (Bohe et al. 2001). On the other hand, protein ingestion results in a rapid increase and subsequent decrease in aminoacidemia that stimulates a maximal postprandial response of MPS in young adults (Moore et al. 2009; Witard et al. 2014). Finally, measurements of mixed MPS have been made (Smith et al. 2011a, b), instead of the myofibrillar fraction that is critical for contractile function. Thus, whether FO supplementation potentiates rates of myofibrillar MPS in response to the oral ingestion of a dose of protein, known to stimulate a maximal response of MPS, remains unknown. In addition, it is unknown whether FO supplementation potentiates rates of myofibrillar MPS when oral protein consumption is combined with resistance exercise. Therefore, the primary aim of this study was to investigate the impact of 8 weeks of FO supplementation on the response of myofibrillar MPS to resistance exercise and protein ingestion. To address this aim, we employed a unilateral single leg resistance exercise protocol that allowed us to separate the influence of FO supplementation on the response of myofibrillar MPS to protein ingestion under resting and post exercise conditions within subject. The secondary aim was to investigate the influence of FO supplementation on the activity of kinases involved in protein feeding and resistance exercise‐induced increases in myofibrillar MPS.

## Materials and Methods

### Participants

Twenty resistance‐trained males were recruited from the University of Stirling and surrounding area to participate in the present investigation. Participant characteristics are displayed in Table 1. Prior to the commencement of the experiment each participant provided written informed consent after all procedures and risks of the study were fully explained in lay terms. All procedures conformed to the standards as outlined in the latest version of the Declaration of Helsinki. Following health screening, participants were excluded if they consumed any form of dietary supplementation or were taking any prescribed medication. The East of Scotland Research Ethics Service (EoSRES, Rec No: FB/12/ES/0005) approved the study procedures.

**Table 1:** Characteristics of participants in each group

Parameter	Fish oil (*n* = 9)	Coconut oil (*n* = 10)
Age (yr)	24 ± 0^a^	21 ± 0
Body mass (kg)	87.0 ± 2.6^a^	80.0 ± 8.2
Lean body mass (%)	77.0 ± 1.3	76.0 ± 1.3
Body fat (%)	20.0 ± 1.5	20.0 ± 1.4
LP 1RM (kg)	143.0 ± 8.0^a^	134.0 ± 7.1
LP/kg/BM	2.13 ± 0.1	2.25 ± 0.1
LE 1RM (kg)	68.0 ± 2.5^a^	60.0 ± 2.5
LE/kg/BM	1.01 ± 0.1	1.01 ± 0.0

yr, years; kg, kilogram; LP, leg press; LE, leg extension; 1RM, one repetition maximum; BM, body mass. Values expressed as mean ± standard error of the mean.

a Denotes significantly higher than coconut oil group (*P *<* *0.05).

### Experimental design

In a randomized, between‐groups, repeated measures design, participants were assigned to either a FO (*n* = 10) or coconut oil condition (CO; *n* = 10). Due to an analytical processing error, one participant from the FO group was removed from statistical analysis (FO; n =* *9). Coconut oil was chosen as a control as coconut oil does not contain any n‐3 or n‐6 PUFAs. Thus, coconut oil will not change the n‐6/n‐3 ratio as would corn oil or another PUFA. Moreover, there is no evidence that coconut oil has any impact on muscle protein metabolism.

During each visit to the laboratory, participants were verbally requested to confirm their pattern of oily fish consumption in an attempt to ensure that changes in free‐living oily fish consumption did not influence muscle lipid profiles during the study. Following baseline testing for single leg 1 repetition maximum (RM) on leg press and leg extension as well as body composition using dual‐energy X‐ray absorptiometry (Lunar iDEXA; GE Healthcare, Hertfordshire, UK), participants reported to the laboratory in the fasted state on two separate occasions. During the initial visit a resting muscle sample was obtained for the assessment of muscle phospholipid fatty acid profiles and also for baseline activity of muscle‐specific anabolic signaling kinases (70 kDa ribosomal protein S6 kinase 1 [p70S6K1], pan protein kinase B [PKB], adenosine monophosphate–activated protein kinase [AMPK]*α*1 and AMPK*α*2). Following baseline measurements, participants consumed 5 g/day of n‐3 PUFA‐enriched FO capsules (providing 3500 mg eicosapentaenoic acid [EPA, 20:5n‐3], 900 mg docosahexaenoic acid [DHA, 22:6n‐3], 100 mg docosapentaenoic acid [DPA, 22:5n‐3] and 0.1 mg vitamin E; Ideal Omega‐3; Glasgow Health Solutions Ltd, Glasgow, UK) for 8 weeks. Compliance to the supplementation protocol was assessed by pill count. Following the end of the supplementation period participants returned to the laboratory to participate in the experimental trial, during which the assessment of myofibrillar MPS was made to examine the influence of FO supplementation on the response of MPS to protein ingestion under resting (FED) and postexercise (FEDEX) conditions using a single leg exercise model. Participants were requested to complete a 3‐day food diary questionnaire for 3 days prior to baseline testing and to repeat this pattern of consumption for 3 days leading up to the experimental trial.

### Experimental trial

**Figure 1:**
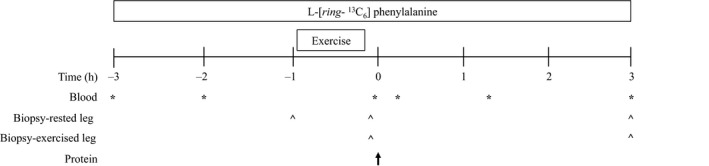
Schematic diagram of the experimental protocol. Initially, a baseline blood sample was drawn followed by a 3‐h resting period. A muscle biopsy was then obtained followed by a bout of high‐intensity unilateral resistance exercise. After the completion of the exercise bout, two muscle biopsies were extracted, one from the exercised leg and one from the rested leg immediately followed by the consumption of 30 g of whey protein. Participants were then rested in a bed for 3 h until a further muscle biopsy was obtained from each leg again.

A schematic illustration of the experimental trial is displayed in Figure 1. On the morning of the trial, participants entered the laboratory at ~0700 h after a 10‐h overnight fast. Each participant then rested in a semisupine position at which time a cannula was inserted into the forearm vein of each arm for blood sampling and l‐[*ring*‐^13^C_6_] phenylalanine (Cambridge Isotope Laboratories, Tewksbury, MA) infusion. After an initial baseline blood sample was drawn a primed, continuous infusion (prime: 2.0 *μ*mol/kg; infusion ~0.05 *μ*mol/kg/min) of l‐[*ring*‐^13^C_6_] phenylalanine was started, the arm heated, and frequent arterialized blood samples obtained. After a 3‐h resting period a resting skeletal muscle biopsy was obtained as described previously (Witard et al. 2014) for the assessment of basal myofibrillar MPS rates and muscle phospholipid fatty acid composition. Following the muscle biopsy, participants performed a bout of high‐intensity unilateral resistance exercise. The exercise bout consisted of 3 sets of 10 repetitions of leg press and leg extension (Cybex International Inc, Cybex International, MA) performed at 70% of individual 1 RM. A 2‐min rest period was allotted between sets and a 3‐min rest period between exercises. After the completion of the exercise bout, two muscle biopsies were performed, one on the exercised leg (FEDEX) and one on the rested leg (FED) immediately followed by the consumption of 30 g whey protein (~0.35 g/kg) diluted in 300 mL of water. Thereafter, participants rested in a bed for 3 h until a further muscle biopsy was obtained from each of the FEDEX and FED legs. All muscle samples were rinsed in ice‐cold saline, blotted to minimize blood saturation of the sample and freed from any visible fat and/or connective tissue. Muscle samples were then frozen in liquid nitrogen and stored at −80°C until further analysis.

### Analytical procedures

#### Blood plasma amino acid concentrations

Plasma amino acid concentrations were determined through use of the Phenomenex EZ:fast amino acid analysis kit with gas chromatography–mass spectrometry (GC Model 6890 Network, Agilent Technologies (Santa Clara, CA); MSD model 5973 Network, Agilent Technologies) as per the manufacturer's specifications.

#### Skeletal muscle phospholipid extraction and analysis

Total lipid content was determined by extraction of lipids from the tissue using 20 volumes of ice‐cold chloroform/methanol (2:1 v/v) using an Ultra‐Turrax tissue disrupter (Fisher Scientific, Loughborough, UK) as reported previously (Folch et al. 1957). Nonlipid impurities were isolated by washing with 0.88% (w/v) KCl and the lower solvent layer containing the lipid extract dried under oxygen‐free nitrogen. The phospholipid fraction was prepared from 0.5 mg of total lipid applied to a 20 × 20‐cm silica gel 60 TLC plate (VWR, Lutterworth, Leicestershire, UK) and developed in isohexane–diethyl ether–acetic acid (80:20:1, by volume) before drying for ~3 min at room temperature. The plate was sprayed lightly with 2,7‐dichlorofluorescein (0.1%, w/v) in 97% methanol (v/v) and the phospholipid bands then were scraped from the plate and placed in a 15‐mL test tube. Fatty acid methyl esters (FAME) were prepared by acid‐catalyzed transesterification in 2 mL of 1% H_2_SO_4_ in methanol at 50°C overnight. The samples were neutralized with 2.5 mL of 2% KHCO_3_ and extracted with 5 mL isohexane–diethyl ether (1:1, v/v) BHT. The samples then were re‐extracted with 5 mL isohexane–diethyl ether (1:1) and the combined extracts were dried and dissolved in 0.3 mL of isohexane prior to FAME analysis. FAME were separated by gas–liquid chromatography using a Thermo Fisher Trace GC 2000 (Thermo Fisher, Hemel Hempstead, UK) equipped with a fused silica capillary column (ZBWax, 60 m × 0.32 × 0.25 mm i.d.; Phenomenex, Macclesfield, UK) with hydrogen as carrier gas and using on‐column injection. The temperature gradient was from 50 to 150°C at 40°C/min and then to 195°C at 1.5°C/min and finally to 220°C at 2°C/min. Individual methyl esters were then identified according to previously published data (Tocher and Harvie 1988). Data were collected and processed using the Chromcard for Windows (version 2.00) computer package (Thermoquest Italia S.p.A., Milan, Italy).

#### Myofibrillar protein synthesis

Myofibrillar MPS was calculated using the precursor–product equation:

$$\text{myofibrillar MPS}=(\left[E_{2b}-E_{1b}\right]/\left[E_{\mathrm{ic}}\times t\right])\times 100$$

where *E*_b_ represents the enrichment of bound myofibrillar protein, *E*_ic_ is the average intracellular enrichment between two biopsies, and *t* is the tracer incorporation time in hours. As we employed “tracer naïve” participants (had not previously participated in a study protocol where l‐[*ring*‐^13^C_6_] phenylalanine was infused), a preinfusion blood sample was used for the calculation of resting myofibrillar MPS (Churchward‐Venne et al. 2012). Myofibrillar and intracellular enrichments of l‐[*ring*‐^13^C_6_] phenylalanine were measured as described previously (Churchward‐Venne et al. 2012). Briefly, for the determination of intracellular enrichments ~25 mg of muscle was homogenized in 0.6 mol/L perchloric acid and the liberated amino acids in the supernatant passed over an ion‐exchange resin (Dowex 50WX8‐200 resin Sigma‐Aldrich, Dorset, UK) and converted to a heptaflurobutyric derivative for analysis using a gas chromatography–MS. For myofibrillar enrichment, ~50 mg of wet weight muscle tissue was homogenized on ice in buffer (10 mL/mg muscle of 25 mmol/L Tris 0.5% v:v triton X‐100 and protease/phosphatase inhibitor cocktail tablets; Complete Protease Inhibitor Mini‐Tabs, Roche, Indianapolis, IN; PhosSTOP, Roche Applied Science, Roche) and centrifuged at 15 000 ***g*** for 10 min at 4°C to separate the supernatant (sarcoplasmic) and pellet (myofibrillar) fractions. The myofibrillar fraction was then hydrolyzed for 72 h in 0.1 mol/L HCl and Dowex (50WX8–200 resin; Sigma‐Aldrich) at 110°C and mixed on a vortex every 24 h. The free amino acids were purified with the use of Dowex ion exchange chromatography, and the *N*‐acetyl‐n‐propyl derivative was prepared and run on an isotope ratio MS to measure the bound enrichment of l‐[*ring*‐^13^C_6_] phenylalanine.

#### Kinase activity

Activity assays were conducted as described previously (McGlory et al. 2014b). Briefly, ~30 mg of human skeletal muscle tissue was homogenized by scissor mincing on ice in RIPA buffer (50 mmol/L Tris/HCl, pH 7.5; 50 mmol/L NaF; 500 mmol/L NaCl; 1 mmol/L Na vanadate; 1 mmol/L EDTA; 1% [vol/vol] triton X‐100; 5 mmol/L Na pyrophosphate; 0.27 mmol/L sucrose; and 0.1% [vol/vol] 2‐mercaptoethanol, and complete protease inhibitor cocktail [Roche]) followed by shaking on a shaking platform for 60 min at 4°C. Debris was removed by centrifugation at 4°C for 15 min at 13 000 ***g***. The supernatant was then removed and protein concentration determined using the BCA protein assay according to the manufacturer's instructions (Sigma‐Aldrich). All assays were carried out by immunoprecipitation either for 2 h at 4°C or overnight at 4°C in homogenization buffer (AMPK [50 mmol/L Tris‐HCl pH 7.25, 150 mmol/L NaCl, 50 mmol/L NaF, 5 mmol/L NaPPi, 1 mmol/L EDTA, 1 mmol/L EGTA, 1 mmol/L DTT, 0.1 mmol/L benzamidine, 0.1 mmol/L PMSF, 5 *μ*g/mL soyabean trypsin inhibitor, 1% (v/v) TritonX‐100] and p70S6K1/panPKB [50 mmol/L Tris‐HCl pH 7.5, 0.1 mmol/L EGTA, 1 mmol/L EDTA, 1% (v/v) tritonX‐100, 50 mmol/L NaF, 5 mmol/L NaPPi, 0.27 mol/L sucrose, 0.1% *β*‐mercaptoethanol, 1 mmol/L Na_3_(OV)_4_, and 1 Complete [Roche] protease inhibitor tablet per 10 mL).

### Statistical analysis

Anthropometric and 1 RM data were assessed using a between‐groups Student's *t* test. All other data were analyzed using two‐factor (treatment; FO vs. CO) repeated measures (time) analysis of variance (ANOVA). When a significant effect of treatment and/or time was detected a Tukey's post hoc analysis was applied to identify where differences existed. Statistical analysis was conducted using statistical package for the social sciences (SPSS) version 18.0 (IBM, Hampshire, UK). Significance was set at *α *< 0.05 and all data are expressed as means ± SEM.

## Results

### Blood plasma amino acid concentrations

**Figure 2:**
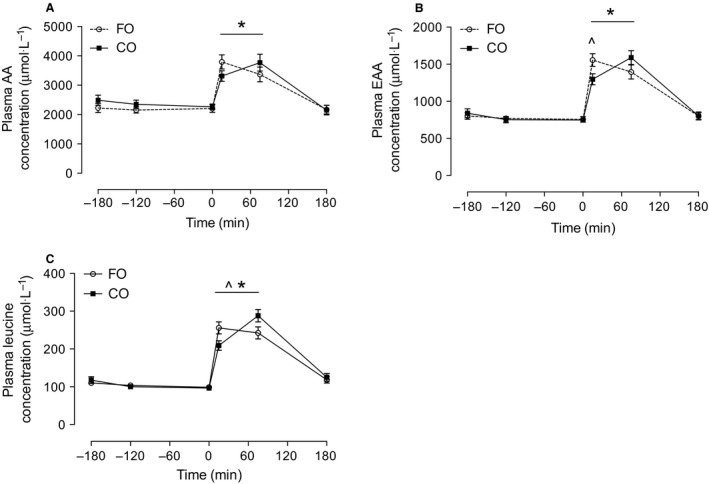
Blood plasma concentrations (*μ*mol/L) of total amino acids (AA) (A), essential amino acids (EAA) (B), and leucine (C). *Denotes significantly different (*P *<* *0.05) from all other time points. ^Denotes significant difference between groups. Data expressed as mean (±SEM).

In both groups, plasma concentrations of total amino acids, EAAs, and free leucine were significantly above baseline at 15 min and 75 min following resistance exercise and protein feeding (*P *<* *0.05) but returned to resting values after 180 min (*P *>* *0.05; Fig. 2A–C). The increase in plasma concentrations of EAAs and leucine in the FO group were significantly higher than the CO group at 15 min postexercise and protein feeding (*P *<* *0.05; Fig. 2B–C), but the plasma concentration of leucine was significantly lower in the FO group compared to the CO group at 75 min postexercise and protein feeding (*P *<* *0.05; Fig. 2C).

### Skeletal muscle phospholipid composition

All phospholipid profile changes in muscle are shown in Table 2. The % n‐3 PUFA of total fatty acids was marginally higher before supplementation in the FO group compared to the CO group (*P *<* *0.05). However, after supplementation there was a ~twofold increase in the % n‐3 PUFA of total fatty acids (*P *<* *0.05), whereas in the CO group % n‐3 PUFA of total fatty acids remained unchanged (*P *>* *0.05). In contrast, % n‐6 PUFA of total fatty acids was significantly lower before supplementation in the FO group compared to the CO group (*P *<* *0.05). Although the % n‐6 PUFA of total fatty acids was significantly lower after supplementation in FO versus CO (*P *<* *0.05), the % n‐6 PUFA of total fatty acids in CO remained unchanged pre–post supplementation (*P *>* *0.05). The % monounsaturated fatty acids of total fatty acids was significantly higher before supplementation in CO compared with FO (*P *<* *0.05), however % monounsaturated fatty acids was reduced after supplementation in CO (*P *<* *0.05) only. There was no significant difference in % saturated fatty acids of total fatty acids between groups before the intervention, however the % saturated fatty acids of total fatty acids significantly decreased after supplementation in both groups (*P *<* *0.05). There was no significant difference in % dimethyl acetals of total fatty acids between groups before the intervention, however were significantly increased after supplementation in both groups (*P *<* *0.05), but to a greater extent in the FO group (*P *<* *0.05).

**Table 2:** Muscle phospholipid fatty acid profile changes

	Fish oil		Coconut oil	
	Before	After	Before	After
Saturated fatty acids				
14:0	0.37 ± 0.01	0.33 ± 0.02	0.32 ± 0.02	0.30 ± 0.02
15:0	0.18 ± 0.01	0.14 ± 0.00	0.15 ± 0.01	0.13 ± 0.01
16:0	18.96 ± 0.33	16.25 ± 0.10	18.87 ± 0.34	16.87 ± 0.38
18:0	14.16 ± 0.25	12.72 ± 0.13	14.10 ± 0.12	12.92 ± 0.17
20:0	0.08 ± 0.01	0.07 ± 0.01	0.08 ± 0.01	0.09 ± 0.02
22:0	0.16 ± 0.01	0.16 ± 0.01	0.14 ± 0.01	0.15 ± 0.20
24:0	0.18 ± 0.02	0.17 ± 0.02	0.17 ± 0.02	0.18 ± 0.03
Total	34.09 ± 0.45^a^	29.83 ± 0.14^b^	33.83 ± 0.36^a^	30.61 ± 0.40^b^
Monounsaturated fatty acids				
16:1n‐9	0.17 ± 0.01	0.19 ± 0.01	0.15 ± 0.00	0.16 ± 0.01
16:1n‐7	0.37 ± 0.02	0.33 ± 0.02	0.42 ± 0.01	0.39 ± 0.02
18:1n‐9	6.05 ± 0.16	4.74 ± 0.20	6.25 ± 0.21	5.92 ± 0.29
18:1n‐7	2.01 ± 0.06	1.86 ± 0.06	1.94 ± 0.06	1.89 ± 0.07
20:1n‐9	0.09 ± 0.01	0.07 ± 0.01	0.09 ± 0.01	0.09 ± 0.01
24:1n‐9	0.20 ± 0.01	0.20 ± 0.01	0.22 ± 0.02	0.24 ± 0.03
Total	8.89 ± 0.14	7.35 ± 0.24	9.07 ± 0.22^a^	8.69 ± 0.36
n‐6 polyunsaturated fatty acids				
18:2n‐6	26.87 ± 0.59	24.17 ± 0.65	29.19 ± 0.52	28.72 ± 0.60
18:3n‐6	0.08 ± 0.01	0.07 ± 0.01	0.07 ± 0.00	0.09 ± 0.01
20:2n‐6	0.12 ± 0.01	0.11 ± 0.01	0.12 ± 0.01	0.12 ± 0.01
20:3n‐6	1.29 ± 0.04	1.14 ± 0.03	1.30 ± 0.09	1.48 ± 0.09
20:4n‐6	13.55 ± 0.56	13.11 ± 0.38	12.67 ± 0.34	13.79 ± 0.47
22:4n‐6	0.44 ± 0.03	0.30 ± 0.02	0.65 ± 0.03	0.75 ± 0.04
22:5n‐6	0.35 ± 0.02	0.20 ± 0.01	0.39 ± 0.01	0.34 ± 0.02
Total	42.69 ± 0.26^a^	39.08 ± 0.43^b^	44.39 ± 0.37^c^	45.30 ± 0.45^c^
n‐3 polyunsaturated fatty acids				
18:3n‐3	0.25 ± 0.02	0.21 ± 0.01	0.26 ± 0.01	0.24 ± 0.01
20:5n‐3	1.16 ± 0.12	4.46 ± 0.22	0.65 ± 0.05	0.69 ± 0.06
22:5n‐3	1.48 ± 0.06	2.27 ± 0.08	1.29 ± 0.06	1.47 ± 0.06
22:6n‐3	2.64 ± 0.18	4.22 ± 0.23	1.55 ± 0.16	1.79 ± 0.21
Total	5.53 ± 0.30^a^	11.16 ± 0.45^b^	3.74 ± 0.23^c^	4.16 ± 0.31^c^
Dimethyl acetals				
16:0DMA	5.32 ± 0.28	7.59 ± 0.12	5.35 ± 0.12	6.70 ± 0.23
18:0DMA	1.88 ± 0.11	2.68 ± 0.13	1.96 ± 0.07	2.43 ± 0.08
18:1DMA	1.62 ± 0.07	2.312 ± 0.07	1.67 ± 0.08	2.11 ± 0.09
Total	8.81 ± 0.38^a^	12.58 ± 0.13^b^	8.97 ± 0.23^a^	11.25 ± 0.29^c^

Data expressed as % total fatty acids, mean ± SEM. Means that do not share a letter are significantly different.

### Myofibrillar protein synthesis

**Figure 3:**
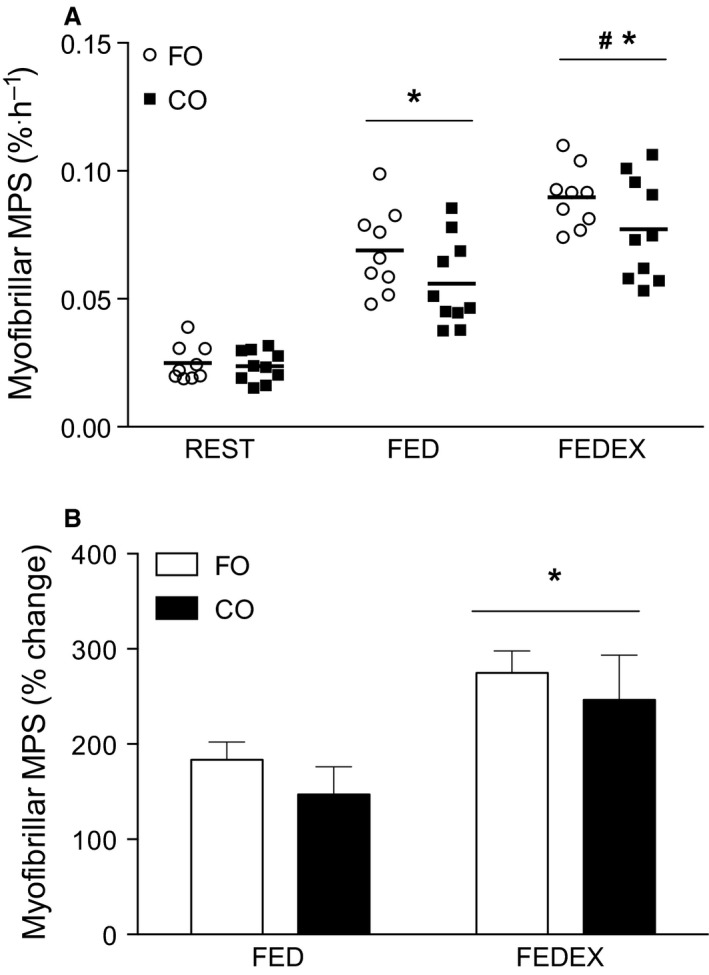
Rates of myofibrillar muscle protein synthesis (MPS) (% per hour) calculated during rest (REST) following protein ingestion (FED) and when protein ingestion was preceded by a bout of resistance exercise (FEDEX). *Denotes significantly different (*P *<* *0.05) from REST (A) and FED (B). ^**#**^Denotes significantly different (*P *<* *0.05) from other time points (A). Data expressed as mean and individual responses for (A) and mean (±SEM) for (B).

The response of MPS was greater in FED compared with REST in both FO (0.025 ± 0.002 to 0.069 ± 0.006% per hour, *P *<* *0.05) and CO (0.024 ± 0.002 to 0.056 ± 0.005% per hour, *P *<* *0.05), however no difference in the feeding‐induced stimulation of MPS was observed between FO and CO (*P *>* *0.05). In FEDEX, MPS was significantly elevated above REST in both FO (0.025 ± 0.002 to 0.091 ± 0.006% per hour, *P *<* *0.05) and CO (0.024 ± 0.002 to 0.077 ± 0.005% per hour*, P *<* *0.05), however, similar to the FED condition, no difference in the feeding plus exercise‐induced stimulation of MPS was observed between FO and CO (*P *>* *0.05; Fig. 3A). There also was no significant effect of supplementation on rates of myofibrillar MPS when expressed as percentage change from REST in FED or FEDEX (*P *>* *0.05; Fig. 3B).

### Kinase activity in response to supplementation

**Figure 4:**
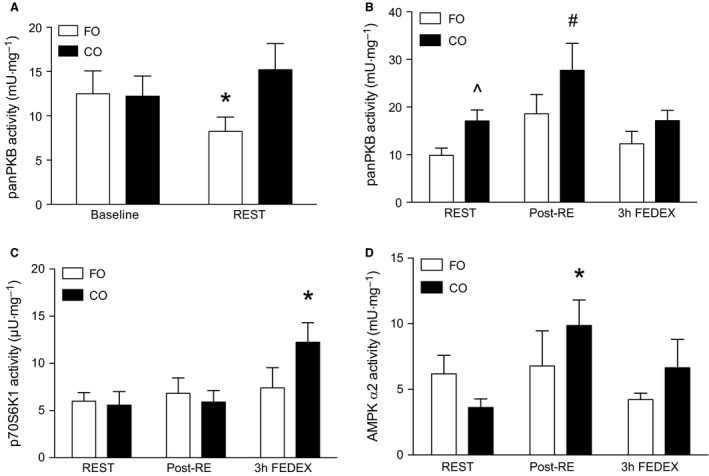
Changes in the activity of panPKB (mU/mg) in response to fish oil (FO) supplementation (A). Changes in the activity of panPKB (mU/mg), p70S6K1 (*μ*U/mg), and AMPK *α*2 (mU/mg) immediately following resistance exercise (post‐RE), and at 3 h following resistance exercise and protein feeding (FEDEX). *^#^Denotes significantly different (*P *<* *0.05) from all other time points, ^^^denotes significantly different (*P *<* *0.05) from the FO group at rest (REST). Data expressed as mean (±SEM).

There were no differences between groups before supplementation in the activity of panPKB, AMPK*α*1, AMPK*α*2, or p70S6K1 (*P *>* *0.05). panPKB activity was significantly suppressed (*P *<* *0.05) at REST compared to before supplementation in the FO group only, indicating that 8 weeks of FO supplementation suppressed basal panPKB activity (Fig. 4A). However, there was no impact of supplementation in either group on the basal activity of AMPK*α*1, AMPK*α*2, or p70S6K1 (*P > *0.05; data not shown).

### Kinase activity in response to protein feeding (FED)

There was no impact of protein feeding on the activity of p70S6K1, AMPK*α*1, AMPK*α*2, or panPKB in either group (*P *>* *0.05; data not shown).

### Kinase activity in response to protein feeding and resistance exercise (FEDEX)

In response to resistance exercise pan PKB activity was significantly increased from REST at post‐RE in the CO group only (*P *<* *0.05; Fig. 4B). P70S6K1 activity was significantly elevated (*P *<* *0.05) at 3 h FEDEX from REST in the CO group (Fig. 4C). However, there was no impact of resistance exercise and protein feeding on p70S6K1 activity post‐RE or at 3 h FEDEX in the FO group (*P *>* *0.05; Fig. 4C). There also was no impact of supplementation on the AMPK*α*1 response to resistance exercise at post‐RE or at 3 h FEDEX (*P *>* *0.05; Fig. 4D). However, in the CO group, in response to resistance exercise, AMPK*α*2 was significantly increased at post‐RE from REST (*P *<* *0.05; Fig. 4D). There was no impact of resistance exercise on AMPK*α*2 post‐RE or resistance exercise and protein feeding at 3 h FEDEX in the FO group (*P *>* *0.05; Fig. 4D).

## Discussion

The novel finding from the present study is that despite a twofold increase in the n‐3 PUFA composition of skeletal muscle, FO supplementation did not significantly enhance rates of myofibrillar MPS at REST nor in either FED or FEDEX condition compared to CO. However, FO supplementation did result in a reduction in resting pan PKB activity, and attenuate p70S6K1 activity at 3 h post‐resistance exercise. As such, these data may suggest that FO supplementation alters anabolic signaling processes, without modulating rates of myofibrillar MPS in response to protein ingestion, or when resistance exercise precedes protein ingestion in healthy, resistance‐trained young males.

Our finding that the rate of myofibrillar MPS was not significantly greater in FED in the FO‐supplemented state is in contrast to previous reports in which 8 weeks of FO supplementation was shown to potentiate rates of mixed MPS in response to a hyperaminoacidemic–hyperinsulinemic infusion (Smith et al. 2011a, b). The lack of agreement between the findings of our study and that of the aforementioned reports (Smith et al. 2011a, b) could be due to differences in muscle fractions assessed as we measured myofibrillar MPS and previous studies (Smith et al. 2011a, b) measured protein synthesis rates of mixed muscle proteins. Additionally, the study population (resistance trained vs. untrained) or the method of amino acid administration (oral vs. intravenous) could also be a contributor to the differences. Indeed, infusion to create a condition of hyperaminoacidemia–hyperinsulinemia as used by Smith et al. (2011a, b) was such that aminoacidemia would be suboptimal for stimulating postprandial MPS. Indeed, in the studies by Smith et al. (2011a, b) plasma leucine concentrations were clamped at ~165–175 *μ*mol/L. In contrast, we provided an oral whey protein bolus of 30 g equating to 0.35 g/kg that has been shown previously to maximally stimulate rates of myofibrillar MPS in young men (Moore et al. 2009; Witard et al. 2014), and in our study resulted in peak plasma leucine concentrations of ~250–300 *μ*mol/L in both conditions. We propose it is possible that the ingestion of 30 g of whey protein in the current study maximized rates of myofibrillar MPS to the extent that FO supplementation would not have exerted a further anabolic influence or was undetectable. In an analogous scenario, our reasoning may explain why the addition of carbohydrate to a saturating protein dose failed to enhance rates of MPS (Staples et al. 2011). We speculate that a “potentiated” MPS response may have been observed if the protein dose our subjects ingested was less than maximally effective.

Similar to the results in the FED condition, we did not observe a significant stimulatory effect of FO supplementation on myofibrillar MPS in FEDEX. Again, we postulate that it is possible that rates of myofibrillar MPS had already been saturated with the combined effect of feeding and exercise (Witard et al. 2014), therefore preventing the detection of any potentiation of myofibrillar MPS with FO supplementation. In contrast to our results, supplementation of elderly women with FO during 12 weeks of resistance exercise training enhanced skeletal muscle strength and functional capacity (Rodacki et al. 2012). Even in the absence of resistance exercise one study has shown 6 months of FO supplementation improves muscle mass and function in elderly men (Smith et al. 2015). We do acknowledge that the present study did not assess changes in muscle strength making a direct comparison between our investigation and others (Rodacki et al. 2012; Smith et al. 2015) difficult. However, it is important to acknowledge that older individuals require a greater amount of protein to maximize rates of MPS compared to young (Yang et al. 2012; Moore et al. 2015), and older adults often fail to consume adequate amounts of protein throughout the day (Fulgoni 2008). Therefore, in these longitudinal studies (Rodacki et al. 2012; Smith et al. 2015) in which protein intake was not controlled, and we speculate, suboptimal, it is plausible that feeding and exercise‐induced rates of MPS were also suboptimal, and thus a potentiation by FO supplementation on MPS and muscle mass was observed.

To examine the impact of FO supplementation on anabolic signaling molecules we employed radiolabeled ^[ϒ−32P]^ATP kinase assays for AMPK*α*2, pan PKB, and p70S6K1 (McGlory et al. 2014b) that is a quantitative readout of kinase activity. Using this method we show that 8 weeks of FO supplementation suppressed the activity of pan PKB at rest as well as AMPK*α*2 immediately following exercise, and p70S6K1 3 h postexercise and feeding. Since the PKB‐mTORC1‐p70S6K1 signaling axis has been shown to be a key phosphorylation cascade regulating MPS (Drummond et al. 2009; Dickinson et al. 2011), our finding of suppressed pan PKB and p70S6K1 activity without a concomitant reduction in rates of myofibrillar MPS may be considered surprising. Indeed, studies have shown that FO supplementation induces alterations in anabolic signaling phosphorylation parallel to changes in MPS in humans following amino acid infusion (Smith et al. 2011a, b) and muscle size in rodents in response to immobilization/remobilization (You et al. 2010a, b). However, a dose–response study in rodents has shown that only a small fraction of p70S6K1 phosphorylation is required to maximize rates of leucine‐feeding‐induced increases in MPS (Crozier et al. 2005). Moreover, other workers have shown that in humans despite the provision of large amounts of amino acids and insulin, rates of MPS remain high in the face of relatively low levels of p70S6K1 phosphorylation (Greenhaff et al. 2008). Thus, our data could be interpreted to suggest that FO supplementation leads to a shift in the relationship between kinase signaling and MPS. That is, less kinase activity is required to maximize rates of MPS in response to oral protein feeding and resistance exercise. It is also conceivable that the timing of the muscle biopsies may have contributed to the observed disconnect between static measurements of anabolic signaling responses, and the dynamic measurement of MPS; both theses remain speculative and warrant further investigation.

The main strength of our study is that we applied the physiologically relevant stimulus of oral protein feeding and resistance exercise rather than intravenous amino acid delivery to stimulate myofibrillar MPS. In addition, the present study is one of few studies in humans that have employed a direct measure of kinase activity in conjunction with the dynamic measurement of myofibrillar MPS, as opposed to semiquantitative immunoblotting. Thus, this study adds important practical information to existing proof‐of‐concept studies that have employed hyperaminoacidemic–hyperinsulinemic infusions (Smith et al. 2011a, b), as well as semiquantitative assessments of kinase activity (You et al. 2010a, b) to examine the impact of FO supplementation on muscle anabolism. However, some limitations of the present study must be acknowledged. Since we elected to assess FED versus FEDEX responses using a unilateral model with a view to minimize the number of muscle biopsies performed (i.e., a between‐subjects design), it is possible that we lacked the necessary statistical power to detect the influence of FO on MPS. For example, the magnitude of change in MPS with FO supplementation in the repeated measures designs of Smith et al. was ~100% in older individuals (Smith et al. 2011a) and ~50% in younger participants (Smith et al. 2011b). In our study, the difference in the change in MPS between FO and CO was smaller (~30 and 35% in FED and FEDEX, respectively). Moreover, these differences were not statistically significant. As a result, we cannot dismiss the possibility that with a greater participant number or a repeated measures design, we may have detected a statistically significant difference between FO and CO supplementation on MPS. However, any potential impact of FO in our study, even if real and undetected, is certainly much less definitive and consistent compared with Smith et al. (2011a, b). Moreover, our measurement of myofibrillar MPS was limited to 3 h postresistance exercise and feeding. Therefore, it is possible that had we extended our capture of MPS to longer than 3 h (i.e., 5 h) we may have detected an effect of FO supplementation. Finally, our participant population was healthy, resistance‐trained young men, and therefore these data cannot be directly extrapolated to females or older adults. Given the relatively larger response in the older than young adults reported by Smith et al. (2011a, b), it would be interesting to repeat our study in an older population. Thus, we emphasize that our data should not be interpreted to conclude that FO supplementation does not potentiate MPS to protein feeding and resistance exercise in all populations, and situations, but rather that these data should be evaluated in the context of the experimental design.

To conclude, we show that 8 weeks of FO supplementation does not significantly enhance rates of myofibrillar MPS in response to ingestion of 30 g of whey protein in healthy, resistance‐trained young men. In addition, FO supplementation did not significantly enhance rates of myofibrillar MPS when the consumption of 30 g of whey protein was preceded by a bout of high‐intensity resistance exercise. Future work examining the impact of FO supplementation in conjunction with resistance exercise training on rates of MPS over a more prolonged period, or in response to suboptimal protein ingestion, in a range of populations may provide further valuable data for the literature.

## Conflict of Interest

None declared.
